# A Nomogram and Risk Classification System Predicting the Prognosis of Patients with De Novo Metastatic Breast Cancer Undergoing Immediate Breast Reconstruction: A Surveillance, Epidemiology, and End Results Population-Based Study

**DOI:** 10.3390/curroncol31010008

**Published:** 2023-12-23

**Authors:** Jingjing Zhao, Shichang Bian, Xu Di, Chunhua Xiao

**Affiliations:** 1 Tianjin Fourth Central Hospital, The Fourth Central Hospital Affiliated to Nankai University, Tianjin 300140, China; 18835570694@163.com (J.Z.); shichangbian2011@163.com (S.B.); 2 The First Department of Breast Cancer, Tianjin Medical University Cancer Institute and Hospital, National Clinical Research Center for Cancer, Tianjin 300060, China

**Keywords:** nomogram, de novo metastatic breast cancer, immediate breast reconstruction, breast cancer-specific survival, SEER database

## Abstract

Background The lifespan of patients diagnosed with de novo metastatic breast cancer (dnMBC) has been prolonged. Nonetheless, there remains substantial debate regarding immediate breast reconstruction (IBR) for this particular subgroup of patients. The aim of this study was to construct a nomogram predicting the breast cancer-specific survival (BCSS) of dnMBC patients who underwent IBR. Methods A total of 682 patients initially diagnosed with metastatic breast cancer (MBC) between 2010 and 2018 in the Surveillance, Epidemiology, and End Results (SEER) database were included in this study. All patients were randomly allocated into training and validation groups at a ratio of 7:3. Univariate Cox hazard regression, least absolute shrinkage and selection operator (LASSO), and best subset regression (BSR) were used for initial variable selection, followed by a backward stepwise multivariate Cox regression to identify prognostic factors and construct a nomogram. Following the validation of the nomogram with concordance indexes (C-index), receiver operating characteristic (ROC) curves, calibration curves, and decision curve analyses (DCAs), risk stratifications were established. Results Age, marital status, T stage, N stage, breast subtype, bone metastasis, brain metastasis, liver metastasis, lung metastasis, radiotherapy, and chemotherapy were independent prognostic factors for BCSS. The C-indexes were 0.707 [95% confidence interval (CI), 0.666–0.748] in the training group and 0.702 (95% CI, 0.639–0.765) in the validation group. In the training group, the AUCs for BCSS were 0.857 (95% CI, 0.770–0.943), 0.747 (95% CI, 0.689–0.804), and 0.700 (95% CI, 0.643–0.757) at 1 year, 3 years, and 5 years, respectively, while in the validation group, the AUCs were 0.840 (95% CI, 0.733–0.947), 0.763 (95% CI, 0.677–0.849), and 0.709 (95% CI, 0.623–0.795) for the same time points. The calibration curves for BCSS probability prediction demonstrated excellent consistency. The DCA curves exhibited strong discrimination power and yielded substantial net benefits. Conclusions The nomogram, constructed based on prognostic risk factors, has the ability to provide personalized predictions for BCSS in dnMBC patients undergoing IBR and serve as a valuable reference for clinical decision making.

## 1. Introduction

About 3–8% of breast cancer patients are detected in stage IV [1]. The 5-year survival rate for early-stage breast cancer patients stands at approximately 96%, while for newly diagnosed metastatic cases, it is around 38% [2]. De novo MBC is typically regarded as incurable, and the primary treatment objectives involve the alleviation of symptoms, the extension of survival, and the enhancement of overall quality of life. In recent years, the prognosis for MBC patients has significantly improved due to the availability of more effective treatment modalities [3]. Mariotto et al. [4] successfully employed a back-calculation method to estimate that between 1992–1994 and 2005–2012, the 5-year relative survival for dnMBC patients aged 15–49 rose from 18% to 36%, marking a twofold increase. The considerably extended lifespan and the rising prevalence of dnMBC patients have heightened the focus on their quality of life [5,6,7]. Breast reconstruction could address physical deficiencies after mastectomy, improve patients’ psychosocial well-being, and enhance their overall quality of life. Studies using both the National Cancer Database and SEER database have demonstrated that more than 10% of metastatic breast cancer patients opt for breast reconstruction [6,7].

Some large-scale retrospective studies have shown that surgical removal of the primary tumor provides a survival benefit for dnMBC patients [8,9,10,11]. Conversely, certain randomized controlled trials have presented opposing research outcomes [12,13,14]. The ESMO guidelines recommend surgical treatment of the primary tumor for dnMBC patients who meet the following criteria: bone-only distant metastasis, hormone receptor-positive, HER2-negative, age < 55 years, have oligometastatic disease (OMD), or with a favorable response to initial systemic therapy [15]. Currently, surgical resection of the primary tumor is not regarded as the standard approach to enhance the survival of dnMBC patients, and there is no consensus on immediate breast reconstruction for this specific patient population. De novo MBC is not a contraindication for breast reconstruction, and as per NCCN guidelines, breast reconstruction after mastectomy is recommended for eligible patients. Nevertheless, given the limited survival of dnMBC patients and the uncertain oncologic safety associated with breast reconstruction, surgeons tend to take a conservative approach in their clinical practice. Durrant et al. [16] conducted a survey in the UK and found that when compared to breast surgeons, plastic surgeons exhibited a higher tendency to opt for IBR in dnMBC patients (53.3% versus 34.7%) and 26.3% of breast surgeons indicated they would refrain from performing any form of breast reconstruction in stage IV cases. Nonetheless, surgeons emphasized that in cases of those with superior response to treatment and extended life expectancy, the proactive choice often leans toward breast reconstruction [16].

The precise identification of which subset of dnMBC patients would significantly benefit from IBR remains unclear; thus, a survival prediction model is needed to assist in the selection of suitable candidates. In this study, we explored prognostic factors in dnMBC patients who underwent IBR based on the SEER database, developed a BCSS prediction nomogram, and externally validated the model. Ultimately, we divided de novo metastatic breast cancer patients into three distinct risk categories—low, intermediate, and high—with the aim of enabling the recognition of high-risk individuals and providing valuable insights for clinical decision making. Transparent reporting of a multivariable prediction model for individual prognosis or diagnosis (TRIPOD) was rigidly referred to in our study [17].

## 2. Methods

### 2.1. Population and Data Collection

**Figure 1:**
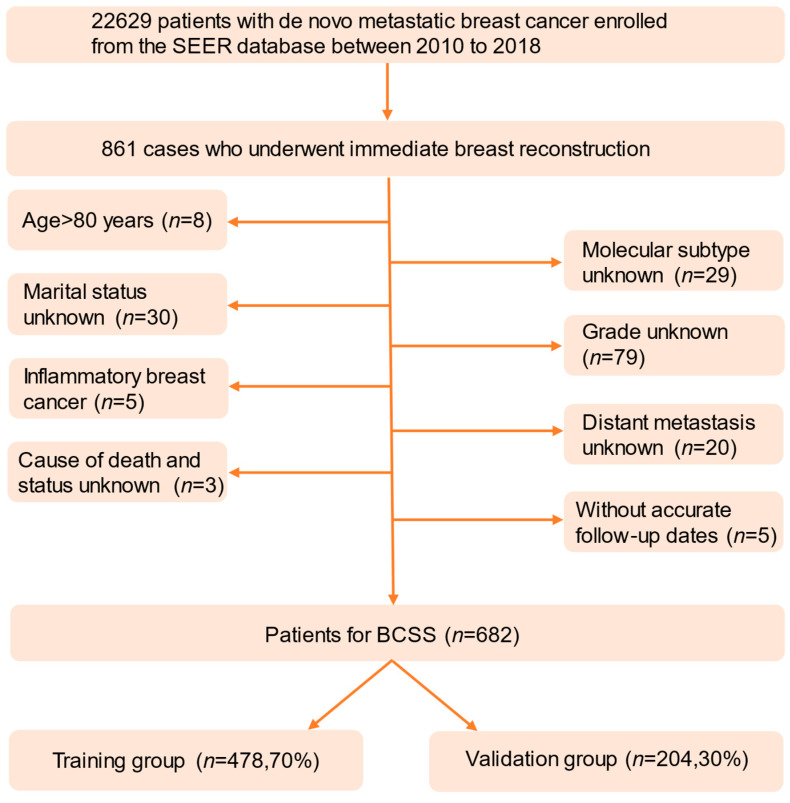
Flow diagram. SEER: the Surveillance, Epidemiology and End Results database, BCSS: breast cancer-specific survival.

Utilizing the SEER*Stat 8.4.1.2 software, we conducted a comprehensive search within the SEER database (http://www.seer.cancer.gov, accessed on 25 July 2023) for female patients diagnosed with dnMBC between 2010 and 2018 who underwent immediate breast reconstruction. The inclusion criteria were as follows: histologically diagnosed as primary breast cancer; de novo metastatic breast cancer; underwent immediate breast reconstruction; age ≤ 80 years; known specific cause of tumor-related death. The exclusion criteria included unknown cause of death; inability to ascertain breast cancer-related mortality; unknown marital status, molecular subtype, histological grade; inflammatory breast cancer; and incomplete survival data. The patients enrolled in this study with complete information were randomly divided into a training group and a validation group at a 7:3 ratio. The training group data contributed to development of the prediction model, while the data in the validation group were employed for validation. The data collection and processing procedures are illustrated in Figure 1.

The information gathered from the SEER database include age at diagnosis, race (white, black, or others), marital status at diagnosis (married or unmarried), laterality (left or right), histologic type (ICD-O-3 8010/3, 8013/3, 8140/3, 8500/3, 8507/3, 8520/3, 8522/3, 8523/3, 8560/3, 8575/3, 8211/3, 8246/3, 8401/3, 8480/3, 8541/3), grade (well, moderate, poor, or undifferentiated), breast subtype, derived AJCC stage 7th edition (2010–2015), derived SEER combined stage group (2016–2017), derived EOD 2018 stage group (2018), surgery of primary site (code with 30, 43–49, 53–59, 63–69, 73–75), radiotherapy and chemotherapy recode, metastasis at distant site, SEER cause-specific death classification (alive, dead of other cause, or death attributable to this cancer), survival time (month), and vital status (alive or dead). X-tile software (version 3.6.1; Yale, New Haven, CT, USA) was employed to determine the optimal age cutoff value. Based on the calculated optimal cutoff value, patients were categorized into two age groups: ≤50 years and >50 years. The primary endpoint of this study was breast cancer-specific survival (BCSS), which was defined as the duration from the diagnosis of dnMBC to the time of death specifically attributable to breast cancer.

### 2.2. Study Design

The initial variables included age, race, marital status, laterality, histologic type, T stage, N stage, ER, PR, HER2, bone metastasis, liver metastasis, lung metastasis, brain metastasis, radiotherapy, and chemotherapy. Preliminary variable selection was conducted on the training group using three different approaches: univariate Cox hazard regression (implemented with the R package: survival), LASSO (utilizing the R package: glmnet), and BSR (employing the R package: leaps). Afterwards, a backward stepwise multivariable Cox regression analysis was performed to determine the final significant independent prognostic factors. In the univariate Cox hazard regression analysis, variables with a *p*-value < 0.1 were eligible for inclusion in the subsequent multivariable Cox regression analysis. LASSO alleviates severe multicollinearity by introducing a penalty function that shrinks variable coefficients to prevent overfitting. The BSR method selects the optimal model under the current variable conditions based on the criterion of adjusted R^2^. In multivariable Cox regression analysis, multicollinearity among variables was assessed using variance inflation factor (VIF) values, and variables with VIF values exceeding 5.0 were eliminated. The optimal predictive model was determined by comparing ROC curves and Akaike information criterion (AIC) values. The ROC curve is a graphical tool that illustrates the performance of a classifier, depicting the relationship between the True Positive Rate (TPR) and False Positive Rate (FPR) at different thresholds. The area under the curve (AUC) is used to measure classifier performance. A higher AUC value, closer to 1, indicates better classifier performance, while a value closer to 0 suggests poorer performance. The AIC value is a generalized information criterion used for comparing the relative goodness of fit of different models and selecting the best model. It achieves a balance between minimizing the sum of squared residuals and penalizing models with more parameters. Therefore, AIC provides a better trade-off by considering the balance between model fit and complexity, with lower AIC values indicating better models. A nomogram for predicting 1-year, 3-year, and 5-year BCSS was generated based on the ultimate multivariable Cox regression model using the “rms” package in R software.

External validation of the nomogram was performed, and ROC curves and AUC values were used to evaluate the discrimination of the nomogram. Further analysis involved the calibration curves (resampling B = 1000) to assess the disparities between the predicted BCSS and actual BCSS in both the training and validation groups. Decision curve analysis was applied to measure the clinical benefits of the nomogram. Using a nomogram approach with the R package, nomogramFormula, we computed the BCSS risk scores for all patients, and then we stratified them into low-, intermediate-, and high-risk groups according to the optimal risk stratification cutoff values determined by the X-tile software (version 3.6.1; Yale, New Haven, CT, USA).

### 2.3. Statistical Analysis

All the statistical analyses were completed with R software (version 4.2.1). Differences in baseline characteristics between the training and validation groups were analyzed through the Chi-square test. Kaplan–Meier curves along with the log-rank test were used to illustrate and compare the BCSS of patients in the distinct risk groups. The median follow-up time was calculated using the reverse Kaplan–Meier method. The hazard ratios (HRs) and 95% confidence intervals (95% CI) of variables were calculated. HRs are primarily applied in survival analysis to estimate the multiple by which the outcome event risk changes in the exposed group compared to the non-exposed group. The confidence interval refers to the estimated range of a population parameter constructed from sample statistics. The 95% confidence interval indicates that there is a 95% probability that the true value of a population parameter will fall within the interval measured by the sample results. A two-tailed *p*-value < 0.05 is considered statistically significant.

### 2.4. Ethics Statement

Before the commencement of our research, we formally submitted a data access application to the SEER database and received an official, authorized license for its use. It remains open and accessible without undergoing ethical review or obtaining informed consent from patients.

## 3. Results

### 3.1. Baseline Characteristics in the Training and Validation Cohorts

This study included a total of 682 de novo metastatic breast cancer patients who underwent IBR with complete data from January 2010 to December 2018. After employing R software for random assignment, the training group consisted of 478 patients, while the validation group comprised 204 individuals. The median follow-up times for the training and validation groups were 67 months and 62 months, respectively. Baseline characteristics of the two groups are presented in Table 1. In the training cohort, 53.3% of patients were in the age group of ≤50 years, while 46.7% were classified as >50 years of age. In the validation group, these percentages were 54.4% and 45.6%, respectively. In both cohorts, the predominant racial group was white, accounting for 77.6% and 71.6%, while the majority of patients exhibited histological characteristics consistent with invasive ductal carcinoma, with proportions of 81.0% and 80.4% in the respective groups. In the training group, there were 270 patients (56.5%) with bone metastasis, 90 patients (18.8%) with liver metastasis, 74 patients (15.5%) with lung metastasis, and 9 patients (1.9%) with brain metastasis. In the validation group, these numbers were 100 (49.0%), 45 (22.1%), 29 (14.2%), and 4 (2.0%). The most common T and N stages were T2 (39.3% in the training group and 43.6% in the validation group) and N2-3 (45.0% in the training group and 50.0% in the validation group). In the training cohort, 242 patients (50.6%) underwent radiation therapy, while in the validation cohort, 105 patients (51.5%) received this treatment. Moreover, chemotherapy was administered to 398 patients (83.3%) in the training cohort and 173 patients (84.8%) in the validation cohort. The *p*-values for the chi-square tests of all variables between the two groups exceeded 0.05.

### 3.2. Variable Selection

**Figure 2:**
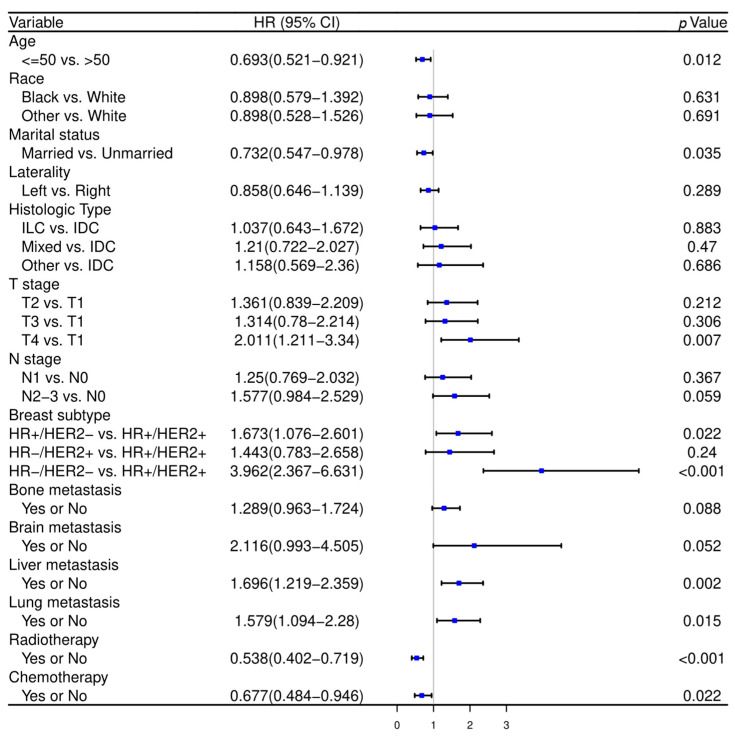
The forest plot of univariate Cox hazard regression in the training cohort. HR—hazard ratio, CI—confidence interval, IDC—invasive ductal cancer, ILC—invasive lobular cancer, HR—hormone receptor, HER2—human epithelial growth factor receptor type 2.

**Figure 3:**
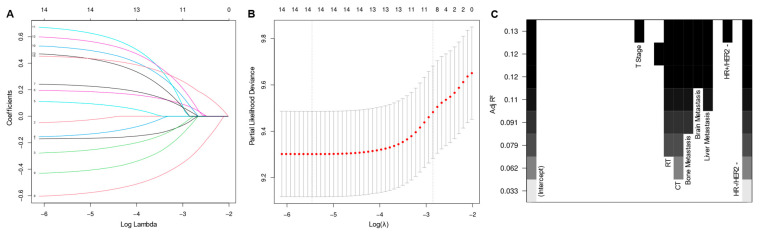
Least absolute shrinkage and selection operator regression (**A**,**B**) and best subset regression (**C**) for initial variable selection. RT—radiotherapy, CT—chemotherapy, HR—hormone receptor, HER2—human epithelial growth factor receptor type 2.

**Figure 4:**
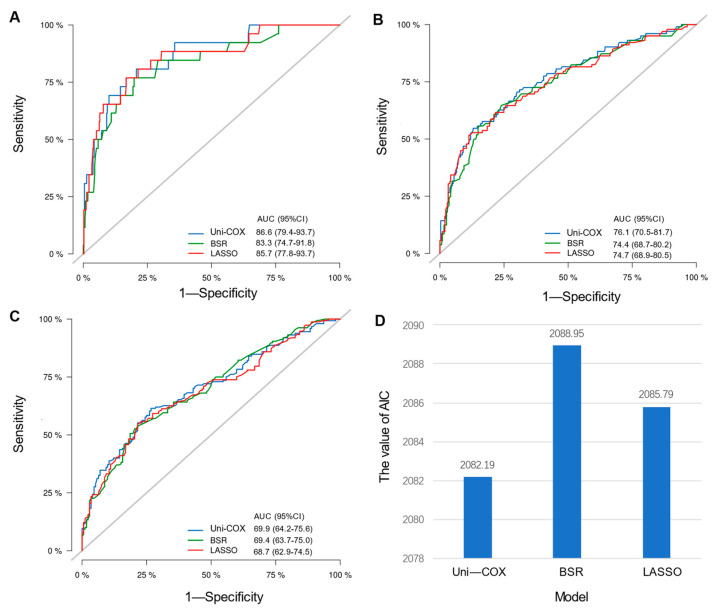
Comparison among different models. (**A**) Comparison of AUC values for the 1-year ROC curves among Uni-cox, LASSO, and BSR models; (**B**) comparison of AUC values for the 3-year ROC curves among three models; (**C**) comparison of AUC values for the 5-year ROC curves among models; (**D**) comparison of AIC values among models.

Initial variable selection was conducted through the application of univariate Cox hazard regression, LASSO, and BSR methods. As shown in Figure 2, univariate Cox hazard regression identified 11 variables with *p*-values < 0.1, including age, marital status, T stage, N stage, breast subtype, bone metastasis, brain metastasis, liver metastasis, lung metastasis, radiotherapy, and chemotherapy. In the LASSO regression analysis, we identified nine variables associated with the lambda.1se value (0.05757954), comprising age, marital status, T stage, N stage, radiotherapy, chemotherapy, bone metastasis, liver metastasis, and breast subtype (Figure 3A,B). In the BSR analysis, the maximum adjusted R^2^ value observed was 0.130, leading to the final selection of seven variables: T stage, radiotherapy, chemotherapy, bone metastasis, liver metastasis, brain metastasis, and breast subtype (Figure 3C). The variables derived from each regression approach were subsequently subjected to a backward stepwise multivariable Cox regression analysis, culminating in the formulation of three predictive models, as presented in Table 2. Following analysis using R software, the predictive model established through multivariate Cox regression of variables initially selected by univariate Cox regression achieved the highest AUC values for the 1-year, 3-year, and 5-year ROC curves (Figure 4A–C). Furthermore, it yielded the lowest AIC value (AIC = 2082.19) (Figure 4D). The optimal predictive model was determined with the following variables: age, marital status, T stage, N stage, breast subtype, bone metastasis, brain metastasis, liver metastasis, lung metastasis, radiotherapy, and chemotherapy.

### 3.3. Development and Validation of a Predicting Nomogram

**Figure 5:**
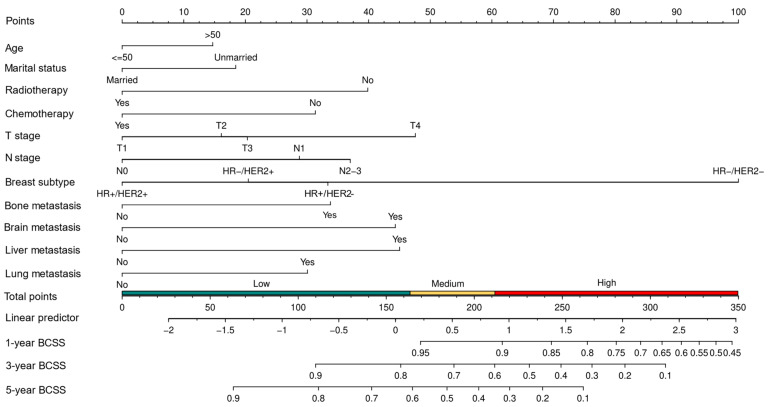
Nomogram to predict 1-, 3-, and 5-year of BCSS for de novo metastatic breast cancer patients with immediate breast reconstruction. *BCSS*—breast cancer-specific survival.

**Figure 6:**
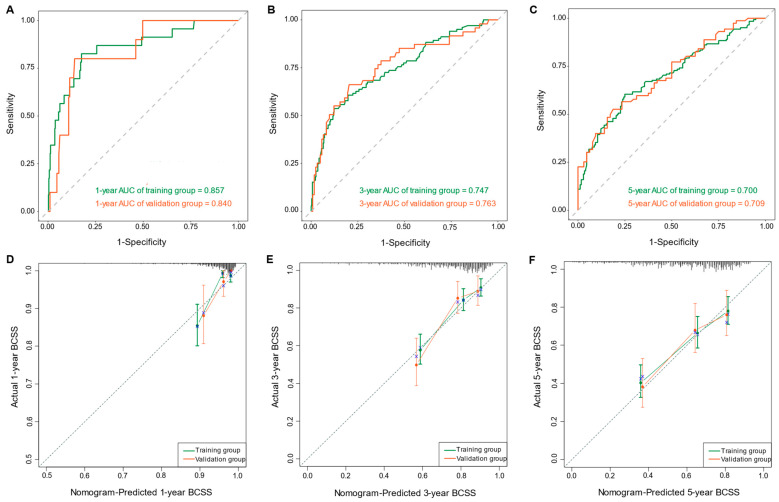

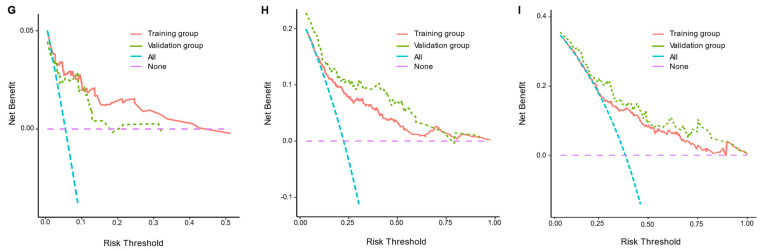
The time-dependent ROC curves of the nomogram predicting (**A**) 1-year BCSS, (**B**) 3-year BCSS, and (**C**) 5-year BCSS of the training and validation groups, respectively. The calibration curves of the nomogram for predicting (**D**) 1-year BCSS, (**E**) 3-year BCSS, and (**F**) 5-year BCSS of the training and validation groups, respectively. The decision curve analysis of the nomogram for predicting (**G**) 1-year BCSS, (**H**) 3-year BCSS, and (**I**) 5-year BCSS of the training and validation groups, respectively.

Based on the multivariate Cox regression model, we constructed a nomogram to predict 1-, 3-, and 5-year BCSS (Figure 5). The nomogram was employed to convert the regression hazard coefficients of each variable into a percentage-based scoring system, where higher scores corresponded to a higher risk of mortality for the respective variables. The nomogram in this study reveals that breast cancer subtype exerted the most pronounced influence on patients’ BCSS. Triple-negative breast cancer (TNBC) carried the highest risk with a potential score of 100 points, followed by the hormone receptor-positive, HER2-negative subtype. The triple-positive breast cancer subtype was associated with the lowest risk. The C-indexes of the predictive model in the training and validation cohorts were 0.707 (95% CI, 0.666–0.748) and 0.702 (95% CI, 0.639–0.765), respectively. To assess the discriminatory capability of the nomogram regarding the endpoint event, time-dependent ROC curves were generated using the R software for validation. In the training cohort, ROC curves showed that the area under the curves (AUCs) of the nomogram for predicting 1-, 3-, and 5-year BCSS were 0.857 (95% CI, 0.770–0.943), 0.747 (95% CI, 0.689–0.804), and 0.700 (95% CI, 0.643–0.757), respectively. In the validation cohort, the corresponding AUCs were 0.840 (95% CI, 0.733–0.947), 0.763 (95% CI, 0.677–0.849), and 0.709 (95% CI, 0.623–0.795) (Figure 6A–C). The calibration curve plots in both cohorts demonstrated strong concordance between the predicted BCSS and the actual BCSS, affirming the high accuracy of the model (Figure 6D–F). Additionally, the DCA curves indicated that the nomogram provided greater net benefits compared to both “all” and “none” (Figure 6G–I).

### 3.4. Development of Nomogram Risk Stratification Prediction Model

**Figure 7:**
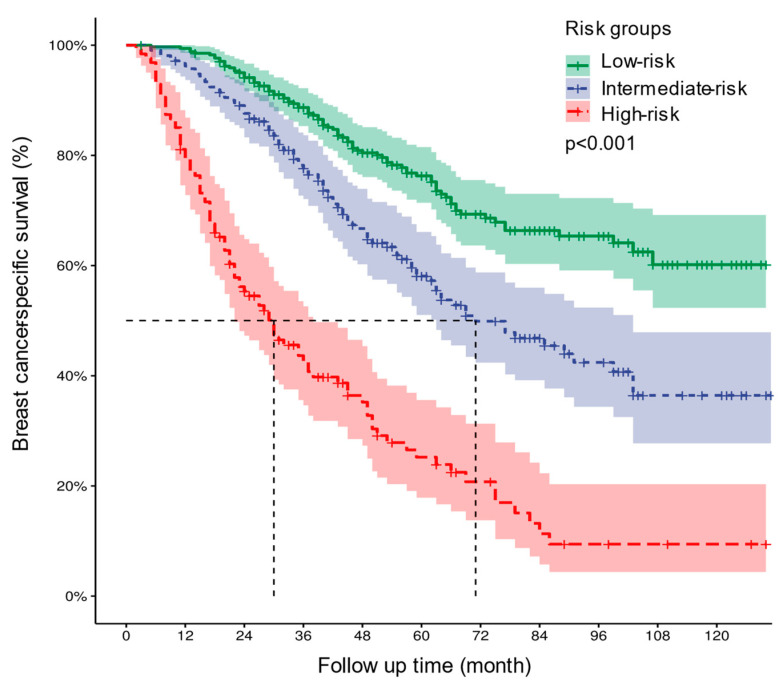
Kaplan–Meier curves of BCSS for all patients in the low-, intermediate-, and high-risk groups. *BCSS*—breast cancer-specific survival.

The risk stratification prediction model was developed based on the points of each patient in the training cohort calculated by the nomogram to divide all patients into three prognostic groups (points < 163—low risk, 163 ≤ points < 212—intermediate risk, and points ≥ 212—high risk). The Kaplan–Meier survival curves showed that BCSS among patients belonging to diverse risk groups was accurately differentiated by the risk stratification prediction model (*p* < 0.001) (Figure 7).

## 4. Discussion

Traditionally, surgical intervention at the primary tumor site has been employed as a palliative strategy to manage localized symptoms in patients with metastatic breast cancer, including issues like ulceration, bleeding, or pain. Based on prior research, the excision of the primary tumor has been associated with a mortality reduction ranging from 18% to 37% [18]. The impact of breast surgery on the survival of dnMBC patients has yielded inconsistent conclusions across several retrospective studies and randomized controlled trials [9,10,12,14,19]. Consequently, there remains an ongoing debate regarding the role of primary tumor resection as a standard therapeutic approach for dnMBC patients. Nevertheless, in recent years, a higher proportion of newly diagnosed stage IV breast cancer patients have chosen to undergo breast surgery [20]. Certain dnMBC patients, driven by the desire for improved breast aesthetics or body image, are more likely to opt for immediate breast reconstruction. Immediate breast reconstruction, which involves the concurrent procedures of mastectomy and implantation of prosthetic devices or autologous tissue transplantation, may potentially increase postoperative complication rates due to the introduction of foreign materials or the more extensive trauma associated with autologous flap transplantation surgery. This may result in delays in postoperative systemic treatments, posing potential risks to oncologic outcomes. However, a study conducted by researchers from the United States analyzed postoperative complication rates in breast reconstruction patients across various disease stages [5]. The findings indicated that there were no statistically significant differences observed between groups in terms of minor, major, or overall complications [5]. Furthermore, the research revealed that breast reconstruction can substantially improve the quality of life for dnMBC patients [5]. Similar results were observed in a study led by Asaad et al. [21], where dnMBC patients who underwent reconstruction exhibited comparable complication rates to those who did not, and there were no treatment delays associated with any of the complications. The study conducted by Wu et al. [6] elucidated that, in dnMBC patients, IBR does not exert any influence on patients’ survival when compared to mastectomy. Immediate breast reconstruction in a dnMBC patient population represents a complex situation marked by a delicate balance of advantages and risks, and it is not universally applicable to all patients. Although prognostic tools already exist for those who have undergone primary tumor surgery [22,23,24], there is still a lack of more accurate prognostic estimates for this population undergoing IBR.

In this study, we analyzed clinicopathological and treatment characteristics of dnMBC patients who underwent IBR using data from the SEER database. We employed three methods, including univariate Cox regression, BSR, and LASSO, to progressively establish a backward stepwise multivariate Cox regression model, with the aim of avoiding overfitting and underfitting. After comparing the AUC and AIC values of various models, we ultimately constructed a predictive model based on variables identified through univariate and multivariate Cox regression, presenting it as a nomogram for reference by surgeons. Then, we conducted a rigorous validation of the nomogram using diverse statistical methods and the model demonstrated excellent predictive performance for BCSS. In addition to the nomogram, this research has devised a risk stratification system that classifies the target population into three prognostic groups, visually integrated into the nomogram for presentation. The prognostic risk prediction model could assist the counseling of patients about reconstruction options and risk versus benefit considerations.

De novo metastatic breast cancer demonstrates a high degree of heterogeneity, manifesting in various aspects such as demographic variables, genetic profiles, molecular subtypes, and patterns of metastasis, leading to considerable variability in the prognostic outcomes of patients [25]. De novo MBC patients with the greatest benefits from surgical intervention were those who underwent treatment with more chemotherapy and those with young age, better economic status, smaller tumor size, fewer positive lymph nodes, lower tumor burden, positive hormone receptor, and good general condition [6,23,26]. Our study further corroborated the association of these factors with BCSS in dnMBC patients who underwent IBR.

This research indicates a notably superior breast cancer-specific survival among patients aged ≤50 who underwent IBR, in contrast to the population aged >50. Prior studies have demonstrated that among metastatic breast cancer patients who underwent surgery, younger age (HR 0.3; *p* = 0.03) and the absence of comorbidities (HR 0.5; *p* = 0.03) are two independent prognostic factors associated with increased survival [27]. In newly diagnosed metastatic breast cancer, younger patient cohorts significantly differ from their older counterparts in terms of tumor biology, prognosis, clinical management, and survival outcomes [28]. In general, younger individuals tend to have more aggressive characteristics, and a relatively younger age, whether in early or advanced breast cancer, is regarded as an independent prognostic risk factor [29,30,31]. Nonetheless, evidence from real-world studies showed that younger patients diagnosed with metastatic breast cancer experienced a more favorable overall survival outcome in comparison to older patients [25,32,33]. This may be attributed to the fact that younger patients often possess a superior baseline health status or exhibit fewer comorbidities, rendering them more amenable to enduring multiline antitumor therapy. Particularly in the case of HR-positive or HER2-positive patients, a range of innovative targeted therapeutic agents can enable these younger individuals to achieve more effective long-term disease management.

However, patients with TNBC tend to have a poorer prognosis regardless of their age [32,33]. In a study involving 7575 newly diagnosed stage IV breast cancer patients, the median overall survival (OS) for TNBC patients was less than 20 months, whereas for HR+HER2- patients, the median OS extended beyond 40 months [34]. In this study, among the eleven variables included in the nomogram, breast subtype exhibited the highest discriminative power. Specifically, within the four subtypes, TNBC consistently presented the highest prognostic risk, while the HR+HER2+ subtype was associated with the most favorable prognosis. Compared to HR-positive or HER2-positive subtypes, the treatment options for TNBC are relatively limited [35]. Studies have shown that in dnMBC patients, the TNBC subtype exhibits a notable elevation in the expression of breast cancer stem cell markers, which is correlated with poorer treatment responses and shorter progression-free survival (PFS) and OS [36]. The Epidemio-Strategy-Medical Economical (ESME)-MBC (NCT03275311) is a multicenter observational study conducted in France, encompassing 16,702 newly diagnosed metastatic breast cancer patients from 2008 to 2014, which revealed a median OS of 42.12 months (95% CI, 40.90–43.10) for the HR+/HER2- subgroup, 44.91 months (95% CI, 42.51–47.90) for the HER2+ subgroup, and 14.52 months (95% CI, 13.70–15.24) for the HR-/HER2- subgroup, respectively [37]. Moreover, the team noted that improvements in the overall survival of dnMBC patients were confined to the HER2-positive subtype over the course of time [37]. The results from Pons-Tostivint et al. [11] demonstrated that, when compared to patients who did not undergo local regional treatment (LRT), LRT significantly improved OS in HR+/HER2- breast cancer patients (61.6 vs. 45.9 months; *p* < 0.001) as well as in HER2+ cases (77.2 vs. 52.6 months; *p* = 0.008). However, there was no statistically significant impact on the OS of patients with TNBC [11]. These reports were mirrored in our study.

Postmastectomy radiotherapy significantly improves the survival of stage IV breast cancer patients [38]. Recently, researchers from the University of Chicago conducted a large-scale observational study based on the National Cancer Data Base (NCDB) to explore the survival benefit of radiotherapy to breast or chest wall after breast surgery in dnMBC patients [39]. They found that in those with ≤two distant metastatic sites, radiotherapy had a positive impact on patient survival; however, no survival benefit from RT was observed in patients with three or more metastatic sites, indicating that RT primarily provides therapeutic benefits to stage IV patients with a lower tumor burden [39]. The nomogram of this study illustrates that patients who did not receive radiotherapy had a BCSS risk even higher than those who did not undergo chemotherapy. In the training cohort of our study, a significant proportion of patients (83.3%) underwent chemotherapy, leading to an underrepresentation of the prognostic risk for patients who did not undergo chemotherapy within the predictive model. In the case of dnMBC patients, it is crucial to balance the assessment of the survival advantages offered by radiotherapy with the consideration of its negative effects on IBR. Radiotherapy has the potential to increase the incidence of postoperative complications in patients undergoing IBR for early-stage breast cancer. Unfortunately, one of the limitations of this study is the unavailability of data from the SEER database to assess the influence of radiotherapy on the surgical outcomes in patients with dnMBC who have undergone IBR.

The developed nomogram in this study enables a more precise prediction of prognosis risk for de novo MBC patients undergoing IBR. Surgeons can use the scoring criteria within the nomogram to assign scores to each clinical or treatment characteristic of advanced breast cancer patients expressing an intention for IBR. Based on the sum of the patient’s risk scores, corresponding 1-year, 3-year, and 5-year BCSS can be determined. Additionally, the risk stratification model facilitates the identification of high-risk patients. In practical clinical settings, this predictive model serves as a valuable tool, aiding surgeons in making well-informed decisions and preventing the oversight of valuable immediate reconstruction opportunities for certain low- or moderate-risk patients.

This study has several limitations: Firstly, the inherent retrospective nature of this research introduces a potential selection bias. Secondly, details regarding radiotherapy, chemotherapy, and other systemic treatments such as hormonal or targeted therapies was unavailable from the SEER database. Thirdly, data regarding postoperative complications and patient-reported outcomes in cases undergoing IBR could not be obtained from the SEER database. Fourthly, the predictive model established in this study necessitates further validation with external datasets or real-world data.

## 5. Conclusions

Our predictive model could support modifying patients with de novo MBC who received IBR by classifying this population into different prognostic risk groups using a combination of clinicopathologic and treatment characteristics. Validation through various statistical methods proved the great performance of the model. Although future external validation with additional datasets is warranted, the developed nomogram holds promise as a valuable tool for guiding personalized treatment decisions in clinical practice.

## Figures and Tables

**Table 1:** Baseline clinicopathological characteristics and treatment experience of patients in the training and validation groups.

Characteristics	Total (*n*= 682)	Training Group (*n* = 478)	Validation Group (*n* = 204)	*p* Value ^a^
Age, years				0.799
≤50	366 (53.7)	255 (53.3)	111 (54.4)	
>50	316 (46.3)	223 (46.7)	93 (45.6)	
Race (*n*, %)				0.171
White	517 (75.8)	371 (77.6)	146 (71.6)	
Black	95 (13.9)	64 (13.4)	31 (15.2)	
Other	70 (10.3)	43 (9.0)	27 (13.2)	
Marital status (*n*, %)				0.164
Married	433 (63.5)	312 (65.3)	121 (59.3)	
Unmarried	249 (36.5)	166 (34.7)	83 (40.7)	
Laterality (*n*, %)				0.751
Left	349 (51.2)	247 (51.7)	102 (50.0)	
Right	333 (48.8)	231 (48.3)	102 (50.0)	
Histologic type (*n*, %)				0.245
IDC	551 (80.8)	387 (81.0)	164 (80.4)	
ILC	55 (8.1)	43 (9.0)	12 (5.9)	
Mixed	50 (7.3)	30 (6.3)	20 (9.8)	
Other	26 (3.8)	18 (3.8)	8 (3.9)	
T stage (*n*, %)				0.698
T1	91 (13.3)	65 (13.6)	26 (12.7)	
T2	277 (40.6)	188 (39.3)	89 (43.6)	
T3	160 (23.5)	117 (24.5)	43 (21.1)	
T4	154 (22.6)	108 (22.6)	46 (22.5)	
N stage (*n*, %)				0.484
N0	89 (13.0)	64 (13.4)	25 (12.3)	
N1	276 (40.5)	199 (41.6)	77 (37.7)	
N2–3	317 (46.5)	215 (45.0)	102 (50.0)	
Breast subtype (*n*, %)				0.194
HR+/HER2+	142 (20.8)	93 (19.5)	49 (24.0)	
HR+/HER2-	387 (56.7)	273 (57.1)	114 (55.9)	
HR-/HER2+	69 (10.1)	55 (11.5)	14 (6.9)	
HR-/HER2-	84 (12.3)	57 (11.9)	27 (13.2)	
Bone metastasis (*n*, %)				0.088
Yes	370 (54.3)	270 (56.5)	100 (49.0)	
No	312 (45.7)	208 (43.5)	104 (51.0)	
Brain metastasis (*n*, %)				1.000
Yes	13 (1.9)	9 (1.9)	4 (2.0)	
No	669 (98.1)	469 (98.1)	200 (98.0)	
Liver metastasis (*n*, %)				0.387
Yes	135 (19.8)	90 (18.8)	45 (22.1)	
No	547 (80.2)	388 (81.2)	159 (77.9)	
Lung metastasis (*n*, %)				0.760
Yes	103 (15.1)	74 (15.5)	29 (14.2)	
No	579 (84.9)	404 (84.5)	175 (85.8)	
Radiotherapy (*n*, %)				0.906
Yes	347 (50.9)	242 (50.6)	105 (51.5)	
No/Unknown	335 (49.1)	236 (49.4)	99 (48.5)	
Chemotherapy (*n*, %)				0.700
Yes	571 (83.7)	398 (83.3)	173 (84.8)	
No/Unknown	111 (16.3)	80 (16.7)	31 (15.2)	

IDC—invasive ductal cancer, ILC—invasive lobular cancer, HR—hormone receptor, HER2—human epithelial growth factor receptor type 2. ^a^ Chi square test.

**Table 2:** Results of backward stepwise multivariate Cox regression in different models.

	Univariate Cox		BSR		LASSO	
	HR (95% CI)	*p* Value	HR (95% CI)	*p* Value	HR (95% CI)	*p* Value
Age (years)						
≤50	0.796 (0.589–1.077)	0.138			0.749 (0.558–1.006)	0.055
>50	1				1	
Marital status						
Married	0.751 (0.556–1.015)	0.063			0.736 (0.546–0.993)	0.045
Unmarried	1				1	
T stage						
T1	1		1		1	
T2	1.284 (0.784–2.102)	0.321	1.369 (0.839–2.233)	0.208	1.397 (0.854–2.285)	0.183
T3	1.371 (0.802–2.344)	0.249	1.490 (0.878–2.529)	0.140	1.570 (0.926–2.662)	0.094
T4	2.093 (1.228–3.566)	0.007	2.502 (1.479–4.234)	<0.001	2.465 (1.457–4.170)	<0.001
N stage						
N0	1					
N1	1.563 (0.929–2.628)	0.092				
N2–3	1.776 (1.065–2.962)	0.028				
Breast subtype						
HR+/HER2+	1		1		1	
HR+/HER2-	1.679 (1.059–2.662)	0.027	1.668 (1.052–2.644)	0.029	1.615 (1.026–2.544)	0.039
HR-/HER2+	1.374 (0.737–2.561)	0.317	1.352 (0.726–2.518)	0.342	1.251 (0.672–2.327)	0.480
HR-/HER2-	4.717 (2.773–8.023)	<0.001	4.545 (2.686–7.690)	<0.001	4.583 (2.704–7.769)	<0.001
Bone metastasis						
Yes	1.690 (1.225–2.331)	0.001	1.715 (1.245–2.363)	<0.001	1.649 (1.203–2.261)	0.002
No	1		1		1	
Liver metastasis						
Yes	2.011 (1.412–2.864)	<0.001	2.083 (1.450–2.992)	<0.001	2.055 (1.438–2.935)	<0.001
No	1		1		1	
Lung metastasis						
Yes	1.595 (1.072–2.373)	0.021				
No	1					
Brain metastasis						
Yes	1.988 (0.891–4.435)	0.093	2.033 (0.936–4.417)	0.073		
No	1		1			
Radiotherapy						
Yes	0.539 (0.399–0.728)	<0.001	0.543 (0.402–0.734)	<0.001	0.543 (0.402–0.733)	<0.001
No/Unknown	1		1		1	
Chemotherapy						
Yes	0.615 (0.422–0.895)	0.011	0.600 (0.415–0.866)	0.006	0.646 (0.445–0.938)	0.022
No/Unknown	1		1		1	

LASSO—least absolute shrinkage and selection operator regression, BSR—best subset regression, HR—hazard ratio, CI—confidence interval, HR—hormone receptor, HER2—human epithelial growth factor receptor type 2.

## Data Availability

The datasets generated and/or analyzed during the current study are available from the corresponding author on reasonable request.

## Abbreviations

dnMBC	De novo metastatic breast cancer
IBR	Immediate breast reconstruction
BCSS	Breast cancer-specific survival
SEER	Surveillance, Epidemiology, and End Results
LASSO	Least absolute shrinkage and selection operator
BSR	Best subset regression
C-index	Concordance index
ROC	Receiver operating characteristic
DCA	Decision curve analysis
AUC	Area under the curve
AIC	Akaike information criterion
